# Remifentanil improves left ventricular diastolic parameters in patients with impaired diastolic function: a prospective clinical study

**DOI:** 10.1186/s12871-024-02425-9

**Published:** 2024-02-01

**Authors:** Özge Köner, Mustafa Aytek Şimşek, Nurcan Kızılcık, Çiğdem Koca, Ayça Türer Cabbar

**Affiliations:** 1 Anesthesiology Department, Yeditepe University Medical Faculty, Istanbul, Türkiye; 2 Cardiology Department, Yeditepe University Medical Faculty, Istanbul, Türkiye

**Keywords:** Analgesics opioid, Remifentanil; heart, Myocardium; monitoring, Echocardiography, Transthoracic; ventricular dysfunction, Left; ventricular diastolic dysfunction, Left; elderly

## Abstract

**Background:**

Left ventricular diastolic dysfunction has a significant impact on perioperative morbidity and mortality, and its incidence is high in elderly individuals. Anesthetic agents may impair diastolic function, which may increase the incidence of perioperative complications. The aim of this prospective, clinical, phase 4 study was to investigate the effects of remifentanil on left ventricle (LV) diastolic function in patients with diastolic dysfunction. The study was performed on 30 spontaneously breathing subjects (aged 60–80 years) with diastolic dysfunction.

**Methods:**

Thirty patients (aged 60–80 years) with diastolic dysfunction scheduled for surgery were recruited between November 2019 and March 2023. Left ventricle function was evaluated once the intravenous remifentanil infusion reached a target-controlled concentration of 2 ng/ml with transthoracic echocardiography. Analysis of systolic function focused on left ventricular ejection fraction and mean mitral annular S velocity (Sm), whereas diastolic function focused on changes in transmitral peak flow (E), E/A, mitral septal and lateral e’ waves, E/e’ ratios and left atrial volume index following remifentanil infusion.

**Results:**

Diastolic function measures of LV (mitral E/e’, septal and lateral e’ waves) statistically significantly improved (E/e’ from 10.6 ± 2.9 cm.sn^− 1^ to 9.5 ± 2.2 cm.sn^− 1^; *p* = 0.006) following remifentanil infusion. Left atrial volume index decreased following remifentanil infusion without statistical significance (from 55 ± 14.4 ml.cm^− 2^ to 51.6 ± 13.3 ml.cm^− 2^; *p* = 0.1). Systolic function (ejection fraction and Sm) did not change following remifentanil infusion.

**Conclusions:**

Remifentanil improves left ventricular diastolic parameters in patients with preexisting diastolic dysfunction. Our study suggests that remifentanil at a plasma concentration of 2 ng.ml^− 1^ might be used safely in patients with left ventricular diastolic dysfunction.

**Supplementary Information:**

The online version contains supplementary material available at 10.1186/s12871-024-02425-9.

## Introduction

Remifentanil is a new generation opioid with an ultrashort elimination half-life. It shares some of the adverse effects of other opioids such as reduced heart rate and arterial blood pressure during general anesthesia. This hemodynamic disturbance may cause severe cardiovascular instability in some cases [1–4]. Decreased arterial blood pressure, heart rate, cardiac output, and systemic vascular resistance are hemodynamic changes induced by remifentanil [2, 4, 5]. However, the mechanism behind the hypotensive effect of remifentanil is not fully understood. Opioids’ adverse hemodynamic effects may occur either by histamine release or by inhibitory actions on the autonomous and central nervous systems, and result in vasodilation and bradycardia [2–7]. Despite their unfavorable effects on hemodynamics, opioids are thought to be cardioprotective [8, 9]. Remifentanil has been shown to be a safe opioid in regard to its cardiac systolic and diastolic function preserving effects in healthy individuals [10].

Left ventricular (LV) diastolic dysfunction is described as impaired LV relaxation with or without reduced restoring forces (and early diastolic suction), and increased LV chamber stiffness, which increase cardiac filling pressures [11]. Left ventricular diastolic dysfunction has a significant impact on perioperative morbidity and mortality and is frequently overlooked in surgical and critical care patients [12, 13]. The incidence of diastolic dysfunction has been reported to be 12.3% in hypertensive patients according to 2016 guidelines [11]. The occurrence of diastolic dysfunction is also increased in elderly individuals. As the number of elective operations is increasing in elderly individuals, the situation requires attention [14, 15]. However, the mechanism through which diastolic dysfunction increases the risk of postoperative complications is not yet fully understood. It has been speculated that, in patients with diastolic dysfunction, anesthetic substances may lead to impaired hemodynamic function and further impairment of diastolic function, which may be associated with a higher incidence of postoperative complications [16, 17].

To the best of our knowledge, there is no reported study that evaluates the effects of remifentanil on left ventricular function in patients with preexisting diastolic dysfunction. Therefore, the aim of our study was to investigate the effects of a clinically studied concentration of remifentanil [10] on diastolic and systolic left ventricle function in patients with grade 1 or 2 diastolic dysfunction. We hypothesized that remifentanil at a target-controlled concentration of 2.0 ng.ml^− 1^ would not impair the function of the left heart as assessed by transthoracic echocardiography (TTE).

## Methods

Following ethics committee approval (Yeditepe University Hospitals Ethical Committee, İstanbul, Turkey; 15.05.2019-no 1018 and NCT 04117009, chairperson Prof Turgay Çelik, MD) and obtaining written informed consent, 30 individuals undergoing surgical procedures under general anesthesia were enrolled. This research was performed in accordance with the Ethical Principles for Medical Research Involving Human Subjects, outlined in the Helsinki Declaration-2013.

The participants were 60 years or older ASA 1–2 individuals who had grade 1 or 2 diastolic dysfunction. The study is designed as a prospective, single group, phase 4, clinical trial. We began recruiting participants in November 2019 and end in March 2023. The exclusion criteria were history or signs of pulmonary, or cardiac disease (heart failure, tachyarrhythmias, bradyarrhythmias (< 45 per minute), atrial fibrillation, atrioventricular block, severe heart valve disease, acute coronary syndrome, symptoms suggesting cardiac ischemia and left ventricular ejection fraction < 50%), severe renal or hepatic disease, body mass index > 30 kg.m^− 2^, and communication barriers. Patients fasting for more than 8 h were also excluded from the study. Intravenous access was established for all the patients on the ward. No fluid replacement was performed during the 15-minute study period except for the Isolyte-S (Eczacıbaşı, Baxter; İstanbul, Turkey) infusion at a rate of 200 mL per hour along with remifentanil infusion (corresponding to a total fluid infusion of 50–70 ml throughout the study).

Five-lead electrocardiogram including all the extremity leads and V5, pulse oximetry was monitored continuously, and noninvasive arterial pressure was measured every 5 min (Draeger, Medical Systems Inc. Infinity Delta XL, model no: MS14296E5390, MA, USA). Body temperature was measured continuously and kept above 36 ^o^C. Hypertension was defined as an increase of 30% from baseline mean arterial pressure and hypotension was defined as a mean arterial blood pressure < 65 mmHg. A treatment strategy for possible hypotension and hypertension (i.v. boluses of ephedrine (5 mg), and boluses of glyceryl trinitrate (25–50 mg); respectively) was planned. Bradycardia was treated if the heart rate was < 50 per minute, and hypotension was treated if the mean arterial blood pressure was < 65 mmHg. A heart rhythm above 100 per minute was an exclusion criterion. Following the measurement of baseline systemic arterial blood pressure, heart rate, pulse oximetry and Ramsay sedation score these parameters were measured and recorded at 5, 10 min, and immediately before anesthesia induction.

Once the patients were transferred to the operating room, the first (baseline) transthoracic echocardiography **(**TTE) was performed with the patient awake in the partial left lateral position to optimize imaging quality. Following the baseline TTE evaluation, the patient was given oxygen 4 L.min^− 1^ by a facemask, and iv infusion of remifentanil (Ultiva, 2 mg GlaxoSmithKline, İstanbul, Turkey) delivered by a target-controlled infusion system (adjusted to Minto pharmacokinetic model with a plasma mode; Perfusor®Space, BBraun, Melsungen AG, Germany) was started. The target concentration of remifentanil was increased stepwise by 0.5 ng.ml^− 1^. The second TTE was performed as soon as a remifentanil target concentration of 2.0 ng.ml^− 1^ (corresponding to an infusion rate of 0.1 µg.kg.min^− 1^) [10, 18] and stable hemodynamics had been reached. A target concentration of 2.0 ng.ml^− 1^ remifentanil is needed during anesthesia induction in the elderly [19]. Following the second TTE evaluation the study was completed. At the end of the study, patient satisfaction was evaluated using a 5-point score (1 very poor; 2 poor; 3 fair; 4 good; and 5 very good).

### Transthoracic echocardiography

All echocardiographic examinations were performed with a Philips HD 15 ultrasonography system and a Philips S5-2 sector array transducer according to current guidelines [20]. Echocardiographic data of all the patients were recorded and stored for subsequent analysis.

Standard LV parasternal long and short axis views were recorded and two- and four-chamber views were obtained from the apical view. Pulsed-wave Doppler recordings of the mitral inflow were obtained by positioning the sample volume between the tips of the open mitral leaflets using optimal alignment with transmitral blood flow. Isovolumic relaxation time (IVRT) was measured by slightly moving the ultrasound beam toward the LV outflow tract to obtain recordings of both LV inflow and LV outflow signals. The sample volume was placed at the septal and lateral sides of the mitral annulus to record pulsed-wave tissue Doppler imaging (TDI). The acoustic power and the filter frequencies of the system were set to the lowest possible values. During the TTE examination: LV end-diastolic (LVEDV) and LV end-systolic volumes (LVESV), left atrial volume (LAV), peak early (E) and peak late (A) transmitral filling velocities, deceleration time (DT), IVRT, early diastolic velocities (e), and peak systolic velocity (S) of the mitral annulus predefined as the average of the septal and lateral mitral annulus (average e’) measurements obtained by TDI were recorded.

The following derived variables were calculated from the aforementioned data:

LV ejection fraction (LVEF), LV mass, E/A ratio, E/e ratio. Analysis of systolic function focused on LVEF measured by the biplane Simpson method, and analysis of diastolic function focused on E/e. E and e reflect early diastolic filling, which depends on the pressure gradient between the atrium and ventricle, LV myocardial relaxation, and early diastolic untwisting [20]. Diastolic dysfunction was diagnosed and graded according to the American Society of Echocardiography 2016 consensus document [10]. All variables were measured at end-expiration over three preferably consecutive cardiac cycles and averaged by the same experienced physician echocardiographer (M.A.S). The same cardiologist (MAS) interpreted recorded echocardiographic data. Two cardiologists (M.A.S and ATC) evaluated all the patients before inclusion in the study to determine whether there was diastolic dysfunction.

### Statistical analysis

The sample size calculation was based on a preliminary study performed on 12 patients to detect a 15% change in E/e’, and Students’ *t* test revealed that 30 patients were required (α = 0.05 and β > 0.8). Data are presented as the mean ± SD, median or number where appropriate. The Shapiro-Wilk test was performed to check whether continuous variables followed a normal distribution. Quantitative parameters between the groups were compared with independent samples t tests if normally distributed and Mann-Whitney U tests if not normally distributed. Comparisons between the groups were performed with Students’ *t* test or Wilcoxon tests. Comparisons within the groups for repeated and parametric data and nonparametric data were performed with repeated measures ANOVA and Friedman tests, respectively. Univariate linear regression analysis was performed to determine whether sex and diastolic dysfunction were correlated. A *p* value ≤ 0.05 was considered statistically significant. All statistical analyses were performed using SPSS for Windows 25.0 (IBM Corp., Armonk, NY, USA).

## Results

**Fig. 1:**
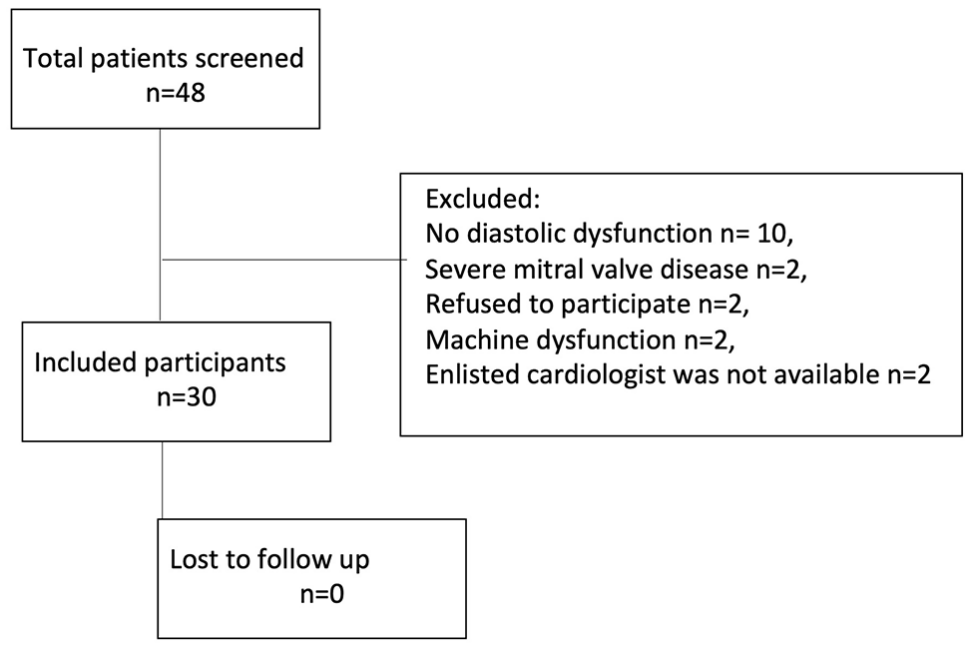
Flow diagram

The demographic and laboratory data of the patients are given in Table 1. Except for two patients with grade-2 diastolic dysfunction, all the recruited patients had grade-1 diastolic dysfunction. None of the patients were lost to follow up during the study period. Of all the patients deemed eligible, 10 did not have diastolic dysfunction, 2 had severe mitral valve disease, and one refused to participate. On two occasions, an enlisted cardiologist was not available. In two cases, evaluation could not be performed due to echocardiography machine dysfunction (Fig. 1).

**Table 1:** Demographic and laboratory data

	n (%)
ASA classification	
I II	1 (3.3) 29 (96.7)
Gender (M / F)	12 / 18 (40 / 60)
Age (years)	70.5 ± 6.9
Height (cm)	164.7 ± 8.6
Body weight (kg)	75.3 ± 8
Body mass index (kg.m^− 2^)	28.2 ± 3.2
Hemoglobin (g.dL^− 1^)	12.6 ± 1.5
Creatinine (g.dL^− 1^)	0.9 ± 0.2
Systemic disease	
Hypertension Diabetes Mellitus	25 (83) 6 (20)

Abbreviation: ASA, American society of anesthesiologists

Systolic, diastolic, and mean blood pressures were significantly decreased 5 min after the initiation of remifentanil infusion compared with the baseline value (*p* = 0.001, *p* < 0.001, and *p* < 0.001; respectively), but remained within the physiologic limits (Table 2). Heart rate was significantly decreased 5 min after the initiation of remifentanil infusion compared with the baseline value (*p* < 0.001; respectively), but the decrement did not significantly change blood pressure and remained within the physiologic range (Table 2). Arterial oxygen saturation monitored with a pulse oximeter was stable and above 95% throughout the remifentanil infusion (Table 2).

**Table 2:** Hemodynamic data, oxygen saturation and sedation scores throughout the study

	Baseline	Remifentanil 5 min	Remifentanil 10 min	Before induction	*p*
SABP (mmHg)	145 ± 15	137 ± 21*	134 ± 24 *	138 ± 19	0.001^a^
DABP (mmHg)	83 ± 11	75 ± 11 *	74 ± 15 *	76 ± 13 *	< 0.001^a^
MABP (mmHg)	112 ± 9	102 ± 14 *	99 ± 16 *	101 ± 12 *	< 0.001^a^
Heart rate (bpm)	73 ± 10	67 ± 11 *	67 ± 8 *	68 ± 8 *	< 0.001^a^
Pulse oximeter (%)	97	96	96	96	ns ^b^
Ramsay score	2 ± 0	2.3 ± 0.5 *	2.7 ± 0.5 *	2.8 ± 0.4*	< 0.001^b^

Abbreviations: SABP; systolic arterial blood pressure, DABP; diastolic arterial blood pressure, MABP; mean arterial blood pressure; ns, not significant. a; repeated measures ANOVA, b; Friedman test. * Statistically significant difference compared to baseline values

The Ramsay score increased significantly following remifentanil infusion and reached a mean value of 2 to a maximum mean value of 3 in all patients (*p* = 0.016). The median patient satisfaction score evaluated using a 5-point score was 4 following remifentanil infusions (Table 2).

### Effects of remifentanil on LV systolic parameters

Baseline systolic function evaluated by LVEF was normal (63.7% ± 6.5) in all patients. The baseline mitral annular S velocity (Sm) was 7.8 ± 1.2 cm.s^− 1^. Remifentanil infusion (at a target concentration of 2 ng.ml^− 1^) did not change LVEF (64% ± 5.7) or mean Sm (7.9 ± 1.2 cm.sec^− 1^; Table 3).

**Table 3:** Echocardiographic data

*n* = 30	Baseline	After Remifentanil	*p*
E (cm.sec^− 1^)	70.3 ± 14.3	73.7 ± 18	0.17
A (cm.sec^− 1^)	88.7 ± 20.4	89.3 ± 23	0.8
E/A ratio	0.8 ± 0.2	0.8 ± 0.2	0.43
Average e’ (cm.sec^− 1^)	6.9 ± 1.3	7.8 ± 1.5*	< 0.001
Septal e’ (cm.sec^− 1^)	6.2 ± 1.6	7.1 ± 1.8*	< 0.001
Lateral e’ (cm.sec^− 1^)	7.6 ± 1.5	8.6 ± 1.5*	< 0.001
E/e’	10.6 ± 2.9	9.5 ± 2.3*	0.006
Mitral annular S velocity (Sm) (cm.sec^− 1^)	7.8 ± 1.2	7.9 ± 1.2	0.6
DT (m.sec^− 1^)	197 ± 48	189 ± 47	0.39
LVEF %	63.7 ± 6.5	64 ± 5.7	0.7
LVESV (ml)	27.7 ± 8	38.2 ± 9.7	0.67
LVEDV (ml)	76 ± 16.5	78 ± 21.5	0.37
LVESD	28.5 ± 3	27.9 ± 3	0.29
LVEDD	45 ± 4.5	45.7 ± 4	0.2
IVSD (cm)	9.9 ± 1.9	10 ± 1.6	0.8
LAVI (ml.m^− 2^)	55 ± 14.5	51.6 ± 13.3	0.1
IVRT (m.sec^− 1^)	99.7 ± 23.7	92.6 ± 25.4	0.08

Abbreviations: DT; deceleration time, LVESV-LVEDV; left ventricle end systolic and, end diastolic volume, LVESD-LVEDD; left ventricle end systolic and, end diastolic dimension, LAVI; left atrial volume index, LVEF; left ventricular ejection fraction, IVRT; isovolumic relaxation time, E; peak early, and A; peak late transmitral filling velocities, e; early diastolic velocities of the mitral annulus predefined as the average of the septal and lateral mitral annulus (average e’), IVSD; left ventricular internal diameter. * Statistically significant difference compared to baseline values

### Effects of remifentanil on LV diastolic parameters

**Fig. 2:**
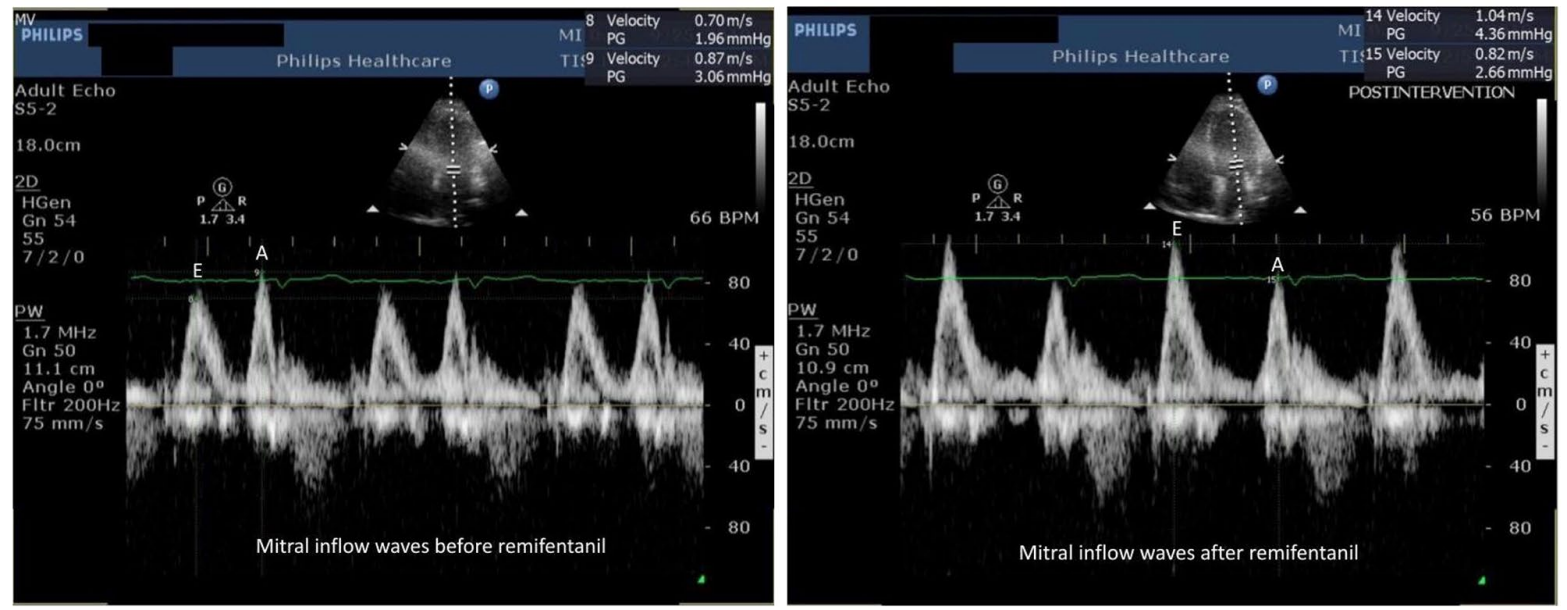
Mitral inflow waves before (left) and after remifentanil (right) Abbreviations: E: transmitral peak early filling velocity, A: transmitral peak late filling velocity

**Fig. 3:**
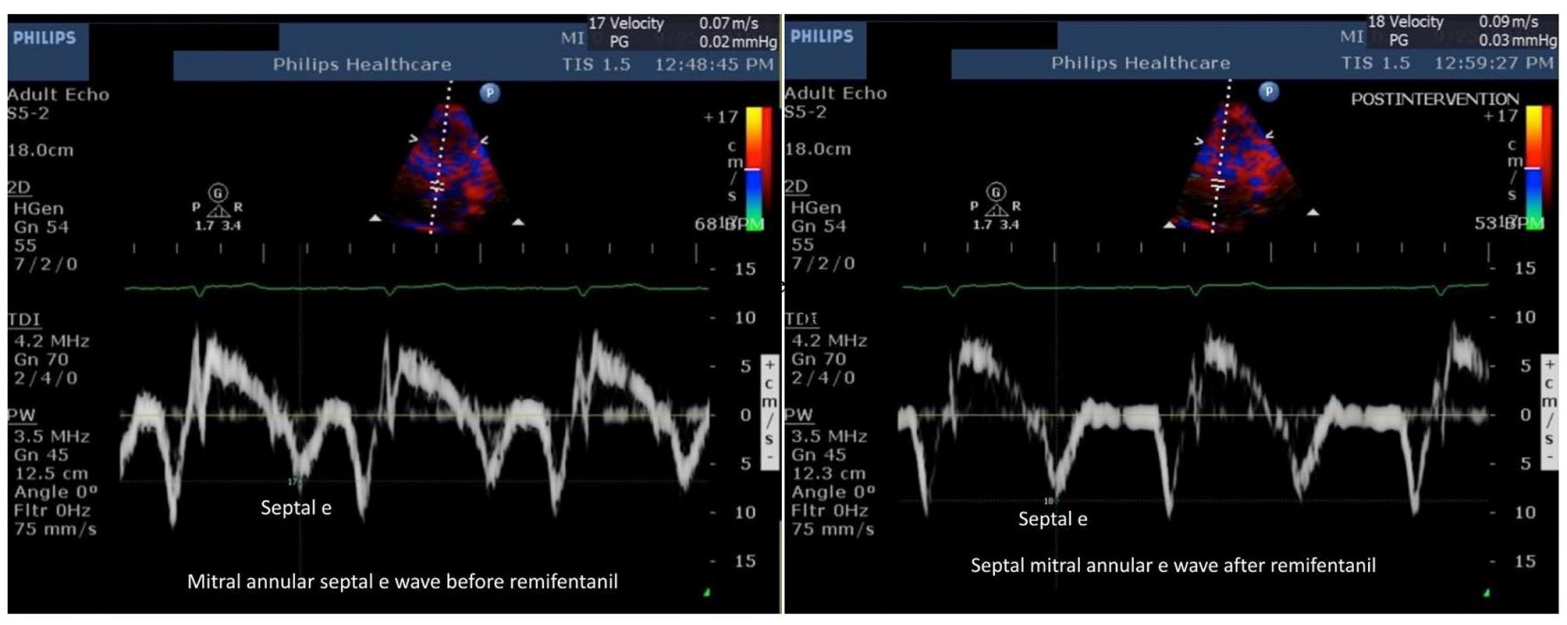
Mitral annular septal e wave before (left) and after remifentanil (right) Abbreviation: e: early diastolic velocity of the mitral annulus

**Fig. 4:**
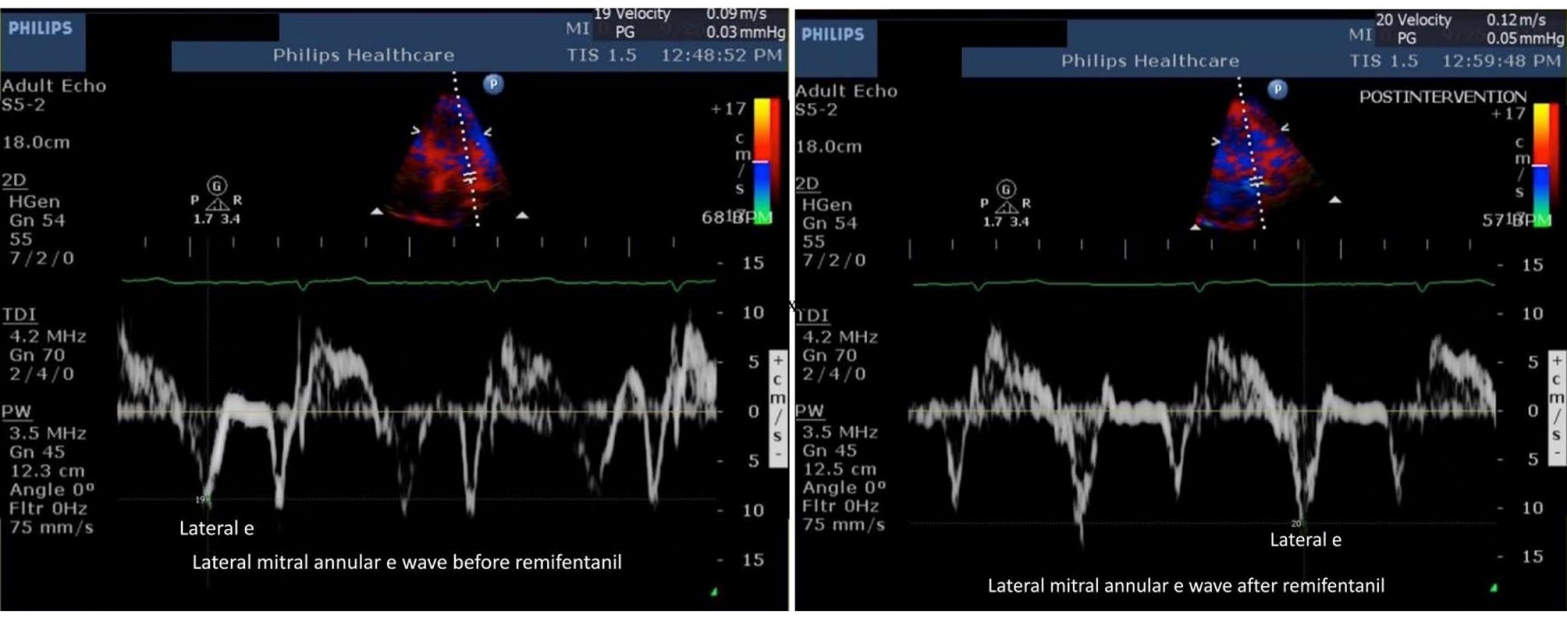
Lateral mitral annular e wave before (left) and after remifentanil (right) Abbreviation: e: early diastolic velocity of the mitral annulus

All recruited patients, except 2 patients who had grade 2 diastolic dysfunction, had grade 1 LV diastolic dysfunction. No change in mitral inflow E, A waves, and E/A ratio was observed following remifentanil infusion (Table 3, Fig. 2). Left atrial volume index (from 55 ± 14.4 ml.cm^− 2^ to 51.6 ± 13.3 ml.cm^− 2^; p = 0.1) and IVRT values decreased from baseline following remifentanil infusion without statistical significance (from 99.7 ± 23.7 msec to 92.6 ± 25.4 msec; p = 0.08). Remifentanil, at a target concentration of 2 ng ml^− 1^, significantly increased the baseline mitral annular septal e’ wave (from 6.2 ± 1.6 cm.sec^− 1^ to 7.1 ± 1.8 cm.sec^− 1^; *p* < 0.001), baseline mitral annular lateral e’ wave (from 7.6 ± 1.5 cm.sec^− 1^ to 8.6 ± 1.5 cm.sec^− 1^; *p* < 0.001), and statistically significantly improved the E/e’ ratio (from 10.6 ± 2.9 cm.sec^− 1^ to 9.5 ± 2.3 cm.sec^− 1^; *p* = 0.006) as shown in Figs. 3 and 4. Deceleration time (DT) decreased from baseline, but without statistical significance (from 197 ± 48 msec to 189 ± 47 msec; *p* = 0.39). End diastolic and systolic volumes (LVEDV, LVESV) did not change during the study (Table 3). Pulmonary vein velocity could be obtained in only 15 of the 30 patients; and therefore, could not be used as a diastolic function parameter.

### Effects of remifentanil on LV diastolic parameters according to sex

There was no statistical correlation between sex and remifentanil induced mitral E/septal e’ changes (95% CI for B 1.02 (-0.43-2.47); *p* = 0.16).

### Effects of remifentanil on other parameters

A respiratory rate < 10 per minute was the most frequently observed side effect of remifentanil infusion, but it was easily corrected with breathing command in all patients. Another side effect, nausea, was observed in only one patient during remifentanil infusion and was treated with ondansetron. None of the patients developed hypoxemia. There were no other complications such as muscle rigidity, respiratory arrest, or hemodynamic changes, that required treatment with vasoactive agents during the study period (Table 4).

**Table 4:** Side effects related to remifentanil infusion during the study period

	n (%)
Respiratory depression	10 (30)
Nausea	1 (3.3)
Bradicardia	5 (10.6)
Patient satisfaction	4.4 ± 0.57

## Discussion

Our study has shown that 2 ng.mL^− 1^ remifentanil given by means of a target-controlled infusion system corresponding to a rate of 0.065–0.08 µg.kg^− 1^.min^− 1^ did not impair systolic and diastolic LV parameters in spontaneously breathing patients with grade 1–2 diastolic dysfunction. Furthermore, improved LV diastolic parameters were observed after remifentanil infusion.

Opioids produce vasodilation by both depressing vasomotor centers in the brainstem and directly affecting on vessels [8]. Diastolic function is affected by changes in preload, and afterload. The improved left ventricular diastolic parameters with remifentanil observed in our study might be related to vasodilation and reduced afterload, as supported by the reduced systemic blood pressure from baseline. Preload was not affected by remifentanil, as indicated by unchanged left atrial and left ventricular end-diastolic volumes. However, as our study was powered to assess changes in E/e′ to evaluate diastolic function, other echocardiographic indices must be interpreted cautiously. Similar to other opioid agents, remifentanil has sedative properties in addition to its analgesic effect. Despite having slight sedation, which was evaluated with the Ramsay score, all patients remained conscious during remifentanil infusion. However, as we did not assess the depth of sedation using BIS, it is not possible to assess its sedative effect on cardiac performance.

The artificial heart provides a favorable condition to evaluate remifentanil’s isolated effects on systemic circulation, as cardiac output is preload-independent in those patients. In their observational study Quattara et al. showed that remifentanil infusion at doses ranging from 0.1 to 1 µg.kg^− 1^.min^− 1^ induces dose-dependent and significant systemic arterial vasodilation without affecting pulmonary arteries in patients with artificial hearts. Remifentanil has not been shown to have significant effects on capacitance vessels. Therefore, it can be suggested that remifentanil-induced systemic blood pressure drop compared to baseline might occur as a result of systemic arterial vasodilation [8]. On the other hand, remifentanil has provided stable hemodynamics even in patients with severely impaired cardiac function when used as an anesthetic agent [4, 21, 22]. Infusion of remifentanil (0.3 µg.kg^− 1^.min^− 1^) along with a constant rate of propofol infusion has not been shown to impair LV systolic and diastolic function in healthy dogs [23]. In our study, baseline LV systolic function (measured by EF and mitral annular S velocity –Sm-) was within the normal range in all recruited patients and did not change following remifentanil infusion. Therefore, the previously mentioned hemodynamic effects of remifentanil do not seem to stem from changes in left ventricle systolic function. The heart rate is also a factor that affects diastolic function; however, in our study, average heart rate values have remained within normal limits. During the measurement of diastolic parameters, the heart rate was below 60 beats per minute in only 5 patients, but since the heart rate remained at 53 beats per minute and above, no treatment was necessary. Therefore, we believe that heart rate values in the aforementioned range did not affect the diastolic parameters.

Different researchers have studied the effects of anesthetic and sedative agents on diastolic function, and the results revealed that anesthetic and sedative agents generally have no unfavorable effect on diastolic function. General anesthesia induced with intravenous anesthetics, sufentanil, midazolam and pancuronium, has been shown to reduce the LV size and result in changes in the biventricular filling patterns, mainly a decrease in most components of Doppler velocities and an improved diastology in patients with diastolic dysfunction [24]. However, whether this improvement observed after anesthetic induction is related to the loading conditions or positive pressure ventilation is not clear. In addition, concomitant use of midazolam and pancuronium makes it difficult to interpret the sole effect of sufentanil. In spontaneously breathing patients with pre-existing diastolic dysfunction, conscious sedation with either midazolam or propofol does not seem to affect LV diastolic performance [25].

In our study, the lack of administration of a concomitant anesthetic or analgesic provides a better understanding of the sole effect of remifentanil on left ventricle function. The stable loading condition provided by our conservative fluid management (approximately 50 mL during the study period) also prevents the potential confounding effect of volume status on diastolic in our study. Besides, no fluid boluses were given to avoid confusing results about the preload and afterload. End diastolic and systolic volumes (LVEDV, LVESV), which reflect LV filling, did not change during our study.

Halothane and sevoflurane have not been shown to influence LV relaxation while propofol has caused slight impairment in diastolic function in healthy, both spontaneously breathing and mechanically ventilated patients [17]. Another study evaluating diastolic function showed that, desflurane and isoflurane, and most likely sevoflurane have no negative effect on early diastolic relaxation in young subjects without systolic or diastolic dysfunction. In contrast, volatile anesthetics appear to decrease atrial function potentially by impairing late diastolic LV filling [17]. However, data concerning the effect of remifentanil on echocardiographic diastolic parameters in patients with diastolic dysfunction are lacking. Our study provides valuable information regarding this matter. The significant increase in septal and lateral e’ velocity and improved mitral E/septal e’ ratio improves LV diastolic filling. Despite not being statistically significant, improved E waves, and decreased IVRT and LAV index from baseline following remifentanil infusion also supports improved diastology.

The potential factors that might have affected the evaluation of diastolic function, the changes in *P*aCO_2_ and *P*aO_2_, are unlikely to be confounders in our study; because previous studies have found that *P*aCO_2_ did not impair LV diastolic and systolic function, and only hyperoxia may affect cardiac function [26–28]. In our study, pulse oximeter levels did not exceed 97% during the study period, a value that is safe considering hyperoxemia.

### Study limitations

Inability to obtain pulmonary vein flow parameters in some of the patients presents a limitation. The results of this study may not reflect the overall effect of remifentanil on left ventricular function in patients with diastolic dysfunction when used along with other anesthetic agents during general anesthesia. The lack of plasma remifentanil concentration measurements is another limitation of our study. The application of oxygen may have influence on myocardial function, however, the relationship is perioperatively not established yet. This is another limitation at the moment. We applied oxygen to all the patients to keep pulse oximeter value around 96–97%.

## Conclusions

In conclusion, we found that remifentanil at a target effect-site concentration of 2 ng.ml^− 1^ improves left ventricular diastolic parameters while preserving LV systolic function in patients with low grade diastolic dysfunction. The results of our study suggest that remifentanil at a plasma concentration of 2 ng.ml^− 1^ might be used safely in this patient group.

### Electronic supplementary material

Below is the link to the electronic supplementary material.

Supplementary Material 1

## Data Availability

Data are added and uploaded as a supplementary XL file.

## Declarations

### Presentation

Preliminary data were not presented previously: none declared.

## Abbreviations

ANOVA: Analysis of variance
BMI: Body mass index
TTE: Transthoracic echocardiography
LV: Left ventricle
Sm: Mean mitral annular S velocity
DT: Deceleration time
LVESV: Left ventricle end-systolic volume
LVEDV: Left ventricle end-diastolic volume
LVEDD: Left ventricle end-systolic dimension
LVEDD: Left ventricle end-diastolic dimension
LAVI: Left atrial volume index
LVEF: Left ventricular ejection fraction
IVRT: Isovolumic relaxation time
E: Transmitral peak early filling velocity
A: Transmitral peak late filling velocity
e: Early diastolic velocity of the mitral annulus
e’: Average velocity of the septal and lateral mitral annulus
IVSD: Left ventricular internal diameter
